# Case from Prof. Greene’s Clinic

**Published:** 1866-04

**Authors:** Frank S. Abbott

**Affiliations:** Clinical Clerk


*Case from Prof. Greene's Clinic.---Berkshire Medical College.---* Reported by Frank S. Abbott, M. D., Clinical Clerk, and communicated for the Boston Medical and Surgical Journal.

Case I.---*Morbus Coxarius.*---Mary C., aged four years. Was always healthy until about eight months ago, when she began to complain of pain in the left knee, limped a little, was restless and oftentimes feverish at night; lost appetite, flesh and strength. For the last three months has not walked at all, or at least has borne no weight on her limb. For the last four or five months she has complained more or less of pain in the hip, but not severe. She is now pale and emaciated. On examination of the affected limb, it was found smaller than the other, and apparently longer than its fellow. Upon measurement, however, there was no difference. Absence of swelling or tenderness, and freedom of motion at the knee, excluded that joint as the seat of the disease. Deep pressure over the *ileo-femoral* articulation gave her great pain. The muscles about the hip were wasted, giving it a flattened appearance, and the ileo-femoral line was almost entirely obliterated. Pressure on the sole or at the knee gave her much pain. Upon carefully questioning the mother, she remembered that about eight months before, while running, she fell and cried, "Oh mother, I have hurt my hip," but was soon at play again as before.

Prof. G. remarked that this was a typical case of coxalgia. The early pain in the knee, without other evidences of inflammation—this being merely a reflex symptom, and one of the most prominent in the early history of this malady—the marked constitutional disturbance at this time showing that some important organ was suffering, the gradual accession of pain and tenderness at the hip, the flattening, the pain upon pressing the caput femoris against the acetabulum, all pointed unequivocally to the nature of the disease. The elongation of the limb was merely apparent and not real, as ascertained by measuring from the superior spinous process of the alium to the malleolus. It occurs from the tilting of the pelvis, and it is doubtful if the elongation spoken of in the books as the result of intra-capsular effusion ever *really* exists. One point should be especially noted here, namely, the *reported fall* of the child about the time the disturbance of its health was first noticed. Dr. G. said that in a majority of the cases he had seen, some such testimony would be elicited, provided the attention of the friends *was called to it*, and there was no doubt in his mind that in a very great proportion of these cases the disease was local in its origin. The idea generally entertained was, that coxalgia was, merely the local expression of a constitutional fault, which was generally a tubercular diathesis. It is true that these inflammations are most likely to occur in scrofulous subjects, in whom a very slight local injury may awaken serious organic changes; and it is true that inflammation established in the joint of such a person will, uncontrolled, give rise to exudations more or less nearly allied to turbercle; but without arguing the question at length, one single fact was almost sufficient to settle it, and that was, that if by any means the pressure and fraction between the opposing surfaces were removed, both local and constitutional symptoms were relieved; and, if taken early, the *great majority* of patients would recover without *any other treatment*.

This, then, was the great indication in this case. Apply a Sayre's splint, making sufficient extension to separate the head of the bone from the acetabular surface. There is not sufficient contraction of the muscles to prevent this; if so, we should tenotomize them. She will then be free from pain, will sleep quietly, and as soon as she is accustomed to the instrument will walk and run with ease, inasmuch as the weight of the body is now upon the perenæum. She is to have also syrup of iodide of iron, a good, nourishing diet, and to be kept as much as possible in the open air.

The splint was applied, and in six weeks the child came walking easily into the clinical room, looking rosy, and complaining of no pain. Six months later, the splint was discontinued, she having perfectly recovered.

CASE II.—*Encysted Tumor.*—John G. has a swelling just over the parotid gland. The fact that the tumor is movable, circumscribed, pailness, fluctuating, and has been three years growing, and that the general health is impaired, is sufficient to warrant the diagnosis of simple encysted tumor.

A simple incision was made down to the sac, which was enucleated without difficulty. Wound healed by first intention.

CASE III.—*Carcinoma Uteri.*—A. S., aged fifty-five, unmarried. Had one child when sixteen years old; none since. Has had repeated attacks of pneumonia and pleurisy within fifteen years, to relieve the effects of which, as she says, she has worn a seton in the chest for ten years constantly. Says she had "ulceration of the womb" five years ago, which was cured by local treatment. A year ago, began to suffer pain through the pelvis, which has come to be very severe, often of a lancinating character. It is aggravated in urinating. Occasionally a slight bloody discharge. General health fair. An aunt and two sisters died of cancer. Professor Green said, we might have here either an inflamed or displaced uterus, or some morbid growth involving the neck or the body of the organ. If benign, the *probabilities* in this case would be that it was a submucous, fibrous tumor, but the history of the case pointed strongly to cancer. We were not, however, to make a diagnosis until we had made a *thorough* exploration of the parts. The same rule should guide us as in examining other parts of the body, to-wit: *possess yourself of all available evidence.* Without this critical examination and thorough analysis of his case, no man can be an *accurate and reliable* diagnostician or therapeutist in surgical diseases of women.

The patient was taken to the ante-room and examined. Sound passed into the uterus, which measured three and a half inches; the anterior lip nodular and of stony hardness, and whole anterior wall of cervix blended with posterior wall of bladder. Thus the case is clear as one of carcinomatous uterus.

*Treatment.*—Live well; take tinct. ferri muriatis, twenty drops, after each meal; two grains of conium at night to relieve pain, and continue to wear the seton. The discharge from it is to a certain extent eliminative, and may have quite an influence in retarding the progress of the malignant disease.

[TO BE CONTINUED.)

